# A Case Report on Fish Bone Perforating Meckel’s Diverticulum Mimicking Appendicitis

**DOI:** 10.7759/cureus.22693

**Published:** 2022-02-28

**Authors:** Jouhar J Kolleri, Abdirahman M Abdirahman, Ali Khaliq, Ala Abu-Dayeh, Sadia Sajid, Salman Mirza, Abdulrazzaq Haider

**Affiliations:** 1 Clinical Imaging Department, Hamad Medical Corporation, Doha, QAT; 2 Anesthesia, Hamad Medical Corporation, Doha, QAT; 3 Pathology and Laboratory Medicine, Hamad Medical Corporation, Doha, QAT

**Keywords:** computed tomography, perforated meckel's diverticulum, meckel's diverticulum, perforation of meckel's diverticulum, fish bone perforation

## Abstract

Meckel’s diverticulum (MD) is the most frequent congenital abnormality of the digestive tract. Although it is silent, it can rarely come up as a complicated case including but not limited to obstruction, inflammation, and neoplasm. Perforation as a consequence of MD is extremely infrequent and mostly related to foreign objects. We report a case of a 24-year-old man who presented to the emergency department with signs and symptoms suggestive of acute appendicitis. Computed tomography (CT) of the abdomen demonstrated foreign body perforation from a protrusion outside the small bowel. The patient underwent laparoscopy and a fish bone perforating MD was found which was removed.

## Introduction

The inability of the omphalomesenteric duct to regress during the first nine weeks post conception leads to a defect of the gastrointestinal tract known as Meckel’s diverticulum (MD) [1]. It is a type of true diverticulum consisting of all the three layers of the bowel wall and usually arises from the antimesenteric border [2]. MD is seen more in males as compared to females; usual age of clinical presentation is younger than the age of two years. It occurs in about 2% of the population and is mostly asymptomatic, but when symptomatic, it presents with obstruction, hemorrhage, and inflammation amongst others. Perforation of MD by foreign bodies is extremely rare because they pass through the alimentary tract without significant consequences [3]. We present a case of a perforated MD due to a fish bone in a young patient which was surgical removed.

## Case presentation

A 24-years-old gentleman came to the emergency department with complaints of lower abdominal pain of one-day duration. The pain was colicky in nature, and not related to food intake. It was associated with constipation and generalized weakness. No history of nausea, vomiting, fever, dysuria was noted.

His vitals were as follows - temperature: 37-degree Celsius, heart rate: 78 per minute, respiratory rate: 16 per minute, blood pressure: 107/70 and oxygen saturation: 99%. On general examination, the patient was conscious and oriented. Abdomen examination showed tenderness in the right lower quadrant pain with guarding, rebound tenderness, and positive Rovsing’s sign. Other systemic examinations were within normal limits. Laboratory examination revealed increased white blood cells and C-reactive protein.

**Figure 1:**
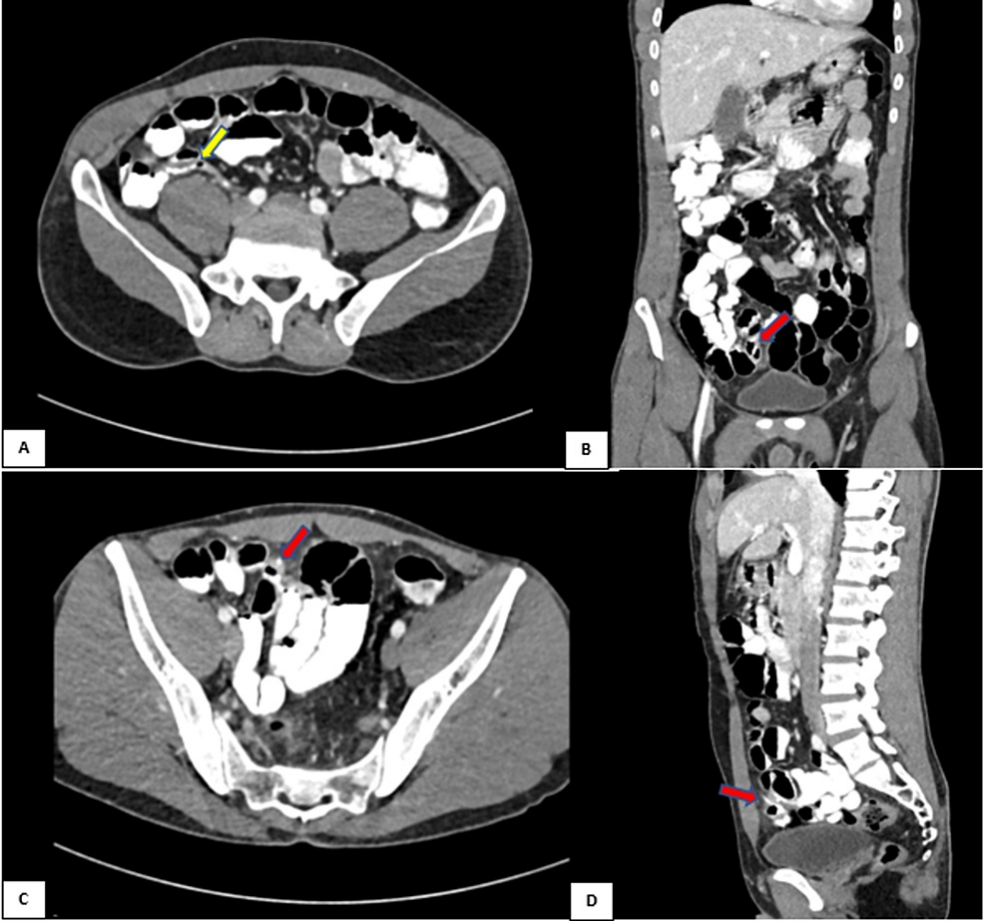
A) Axial CT abdomen and pelvis with oral and IV contrast showing appendix of normal calibre (yellow arrow) with no periappendiceal fat stranding. B) Coronal, C) Axial, and D) Sagittal cuts showing foreign body (fish bone) perforating the distal ileal loop (red arrows).

Computed tomography (CT) of the abdomen and pelvis with oral and intravenous contrast was done to rule out acute appendicitis which revealed an appendix of normal caliber with no significant periappendiceal fat stranding. There was a linear hypodensity measuring 14 mm seen in the right lower quadrant in one of the distal ileal loops and it appeared to be partially protruding outside the bowel wall with mild fat stranding. The possibility of a foreign body could not be excluded. However, there was no obvious adjacent collection seen (Figure 1).

**Figure 2:**
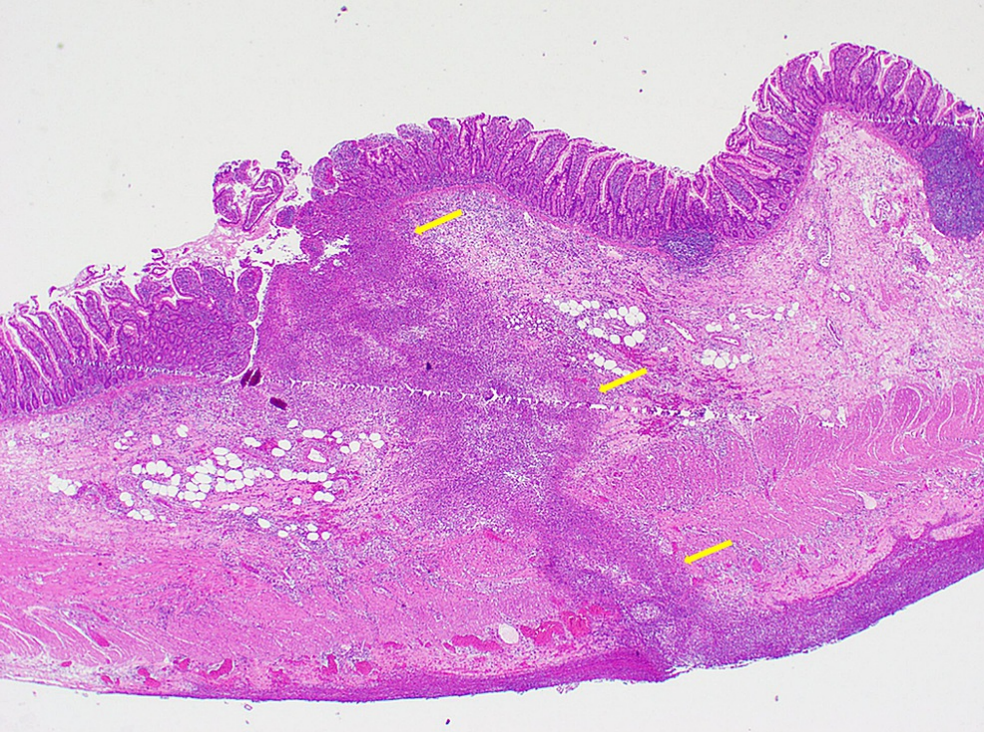
Low-power microscopic view demonstrating small bowel wall with severe transmural acute inflammation and serositis (yellow arrows), consistent with perforation (hematoxylin and eosin stain (H&E), original magnification ×40).

The patient was taken to the operation theatre and underwent laparoscopic exploration. A running of the bowels was performed and MD was found at around 80 cm from the ileocecal valve, with its tip perforated by a fish bone. The fish bone was extracted and MD was excised giving a postoperative diagnosis of fish bone perforating tip of MD. Histopathology report came as small bowel wall with focal severe transmural acute inflammation and serositis, consistent with perforated MD (Figure 2).

The patient was treated with IV normal saline, IV metronidazole 500 mg, three times daily for five days, and cefuroxime sodium 1.5 gm, three times daily for seven days. The postoperative days were uneventful; he became symptomatically better and was discharged home with surgery outpatient follow up.

## Discussion

MD is defined as an incomplete continuity of the omphalomesenteric duct, which connects the intestine to the yolk sac during embryonic development. It is the most common congenital abnormality of the gastrointestinal tract [4]. It has an incidence of 2% in the population and is usually asymptomatic, with symptoms occurring in about 4%-6% of people [4,5]. However, gastrointestinal bleeding is the most common clinical manifestation of MD in children, while intestinal obstruction is the most common complication in adults. Other complications such as diverticulitis, perforation, enterolith formation have also been reported [4].

Ingestion of foreign bodies is one of the most common causes of symptomatic MD secondary to perforation, which accounts for 0.5% of cases. Multiple foreign bodies have been identified as a cause of MD perforation, with fish bones accounting for 55% of cases [6]. Wearing dentures poses a high risk that small foreign objects can pass unnoticed, as the sensitivity of the palate decreases, which affects the sense; mental retardation and alcoholics are also at risk [6].

The majority of ingested foreign bodies pass through the gastrointestinal without serious events and 1% will require surgery [7]. An ischemic region with a large central concavity is developed when the intestinal mucosa is pricked with sharp pointed objects. The intestinal wall increases its lumen at the point of contact and allows the intestine to move more freely to the offending object. Fish bones, due to their sharp point, cause pathological changes in the intestine, which grips the intestinal wall mucosa and cause necrosis, which leads to the formation of a mucosal band around the fishbone, which hooks it onto the tissue [6]. It is known that Asian countries especially Japan and South Korea consume fish frequently and therefore a high number of complications secondary to the ingestion of fish bones were reported [8].

Depending on the location of the damage, the clinical diagnosis is vague and variable. Acute appendicitis, renal colic, acute diverticulitis, and abdominal colitis can all mimic MD perforation. Our patient presented with pain in the right iliac fossa, which seemed to indicate acute appendicitis clinically. Patients with long-term foreign body impaction may develop recurrent inflammation or chronic inflammatory masses, which can be mistaken for a neoplasm [9]. This may be explained by the lack of specificity in the clinical picture, which is frequently detected fortuitously; the aforementioned consequences may be the sole tell-tale sign, and the physician may overlook it. The lack of awareness of foreign body ingestion and the patient's lack of understanding of the link between foreign bodies and his or her clinical picture also contribute to biased clinical reasoning [10].

Plain film radiography is not reliable in finding the fishbone, since its detection sensitivity in the aero-digestive tract is 32%. Abdominal ultrasound has been shown to be useful in the identification of foreign bodies based on their high reflectivity and variable posterior shadowing but with the increased utility of CT in emergency departments particularly in the evaluation of the acute abdomen condition, abdominal ultrasound has a limited place. Thanks to its high resolution, CT remains the radiological examination of choice for the detection of foreign bodies and their complications. The main radiological features of a fishbone perforation are a thickening of the intestinal wall, fatty deposits, intestinal ileus, ascites, localized pneumoperitoneum, intra-abdominal abscess, and a linear hyperdense structure in the abdominal cavity in the gastrointestinal tract or within a parenchymal organ, often surrounded by inflammatory changes. Note that inflammatory changes can alter the anatomical structure that mimics a neoplasm, so finding a linear hyperdense structure in the center of the lesion is critical. Furthermore, positive oral contrast can mask a foreign body in these situations. Avoiding its administration or delaying it in cases of suspected or possible foreign body ingestion [9,10].

Surgical management is frequently required for MD complications. It depends on the patient's symptoms and the nature and location of the swallowed object. When the base of the diverticulum is macroscopically involved or a tumor is present, a segmental bowel resection with primary anastomosis is considered. In some situations, a simple diverticulectomy with transverse suture appears to be sufficient to prevent stenosis [11]. Because laparoscopic surgery causes less damage than traditional open surgery, it has gradually supplanted the latter. Laparoscopic surgery is currently the recommended procedure [12]. The mean mortality from MD has been reported to be around 6% in various series of operations, with a significant number of deaths occurring in the elderly [6].

## Conclusions

Perforation of MD by fish bone is a challenging diagnosis that should be considered in the differential diagnosis of acute abdominal symptoms, particularly in high-risk patients. The unspecific character of the symptoms and the patient’s unawareness of the ingestion event can lead to confusion with other conditions, such as acute appendicitis. Delay in diagnosis and treatment are associated with an increased risk of morbidity and mortality, thus thorough imaging modalities are required to make an accurate diagnosis.
